# The HCV genome domains 5BSL3.1 and 5BSL3.3 act as managers of translation

**DOI:** 10.1038/s41598-018-34422-7

**Published:** 2018-10-31

**Authors:** Cristina Romero-López, Pablo Ríos-Marco, Beatriz Berzal-Herranz, Alfredo Berzal-Herranz

**Affiliations:** Instituto de Parasitología y Biomedicina López-Neyra, (IPBLN-CSIC), Av. del Conocimiento 17, 18016 Armilla, Granada Spain

## Abstract

The RNA genome of the hepatitis C virus (HCV) encodes a single open reading frame (ORF) containing numerous functional elements. Among these, the *cis*-acting replication element (CRE) at the 3′ end of the viral ORF, has become of increasing interest given its dual role as a viral translation repressor and replication enhancer. Long-range RNA-RNA contacts mediated by the CRE build the structural scaffold required for its proper functioning. The recruitment of different cellular factors, many related to the functioning of the translation machinery, might aid in the CRE-exerted downregulation of viral translation. The present data show that the CRE promotes a defect in polysome production, and hinders the assembly of the 80S complex, likely through the direct, high affinity recruitment of the 40S ribosomal subunit. This interaction involves the highly conserved 5BSL3.1 and 5BSL3.3 domains of the CRE, and is strictly dependent on RNA-protein contacts, particularly with the ribosomal proteins RPSA and RPS29. These observations support a model in which the CRE-mediated inhibition of viral translation is a multifactorial process defined by the establishment of long-range RNA-RNA interactions between the 5′ and 3′ ends of the viral genome, the sequestration of the 40S subunit by the CRE, and the subsequent stalling of polysome elongation at the 3′ end of the ORF, all governed by the highly stable hairpin domains 5BSL3.1 and 5BSL3.3. The present data thus suggest a new managerial role in HCV translation for these 5BSL3.1 and 5BSL3.3 domains.

## Introduction

In positive, single-stranded RNA viruses, such as the hepatitis C virus (HCV), the genomic RNA acts as a template for both translation and replication. While the ribosomes move along the viral mRNA, viral RNA polymerase must initiate the synthesis of the minus strand, thus creating a potential steric hindrance at the 3′ end of the viral genome. This could render viral protein synthesis inefficient and reduce viral RNA production. Translation and replication must therefore be intimately coupled, and should not overlap in time, demanding the precise control of both.

**Figure 1:**
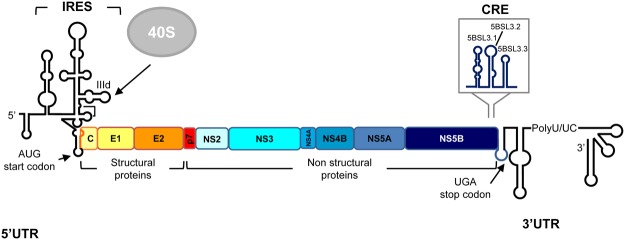
Genetic organisation and functional RNA domains in the HCV genome. Diagram illustrating the genetic organisation of the viral RNA genome, showing the 5′ and 3′ UTRs flanking the single ORF. Viral structural and non-structural (NS) proteins are shown. The minimum region for IRES activity is marked. The 3′ end of the ORF contains the conserved CRE region immediately upstream of the translation stop codon. Translation start and stop codon locations are indicated by arrows.

The HCV RNA genome encodes a single open reading frame (ORF) flanked by highly conserved untranslated regions (5′ and 3′UTR; Fig. 1). During early infection, an internal ribosome entry site (IRES) mapping to the 5′ end of the viral genome directs the initiation of translation by a mechanism different to the canonical cap-dependent method^1,2^. The HCV IRES functions by the direct recruitment of the 40S ribosomal subunit at the essential subdomain IIId in the absence of any other factor^3–7^. Additional, distant, functional genomic RNA domains enhance HCV protein synthesis^8,9^ (Fig. 1). For example, the 3′UTR is in charge of transferring the 40S ribosomal subunit and likely translation initiation factors (e.g., eIF3), from the 3′ to the 5′ end of the mRNA after the translation termination step, thus favouring ribosome recycling^10^.

The precise control of viral protein synthesis is required if competent RNA genomes are to be replicated. In this context, the *cis*-acting replication element (CRE) has been reported to operate as a dual partner, downregulating HCV protein synthesis^11^, and enhancing viral replication^12,13^. The HCV CRE has been characterised as a set of genotypically conserved structural elements, mapping to the very 3′ end of the ORF, immediately upstream of the translation stop codon^12–16^ (Fig. 1). The CRE is defined by three stable stem-loops known as 5BSL3.1, 5BSL3.2 and 5BSL3.3. The central domain, 5BSL3.2, is absolutely indispensable for HCV propagation^12,13,16–18^. The function of domains 5BSL3.1 and 5BSL3.3 remains elusive, although their high sequence and structural conservation suggest their involvement in the control of key steps during the infective cycle.

The mechanism by which the CRE interferes with HCV protein synthesis is not completely understood. It is known, however, that the 5BSL3.2 domain governs a complex network of contacts that organise the conformation of the HCV genome at two levels^19^: (i) by promoting the local structural tuning of the 3′ end of the viral RNA genome^20^, which seems to be essential for replication^16,21^; and (ii) by allowing the circularization of the HCV RNA via the formation of long-range RNA-RNA interactions with subdomain IIId of the IRES region^11,22,23^. The acquisition of a closed-loop topology is a crucial step required for both replication and translation; the circular conformation provides nuclease resistance and creates optimal microenvironments in which different processes can occur. Indeed, mRNA circularization is a strategy adopted by most mRNAs to allow the efficient reloading of any ribosome that reaches the stop codon to the 5′ end of the mRNA.

Our group has previously isolated ribosomal proteins bound to the CRE region^24^. These observations prompted the proposal that the well-known RNA-RNA interaction network involved in the control of viral translation might be aided via the direct recruitment, or sequestration, of the cellular translation machinery. The present work provides evidence for the direct interaction of the CRE with the 40S ribosomal subunit. This interaction is dependent on RNA-protein contacts, especially with the ribosomal proteins RPSA and RPS29, both of which are involved in the efficiency of IRES-mediated viral protein synthesis^25,26^. The present results support a model in which the CRE region sequesters 40S subunits, preventing efficient ribosome reloading at the 5′ end of the mRNA genome, thus repressing translation.

## Results

### The CRE 5BSL3.2 domain interferes with polysome assembly

The translational competence of an mRNA molecule is related to the efficiency with which it recruits ribosomes. The final number of bound ribosomes depends on the length of the ORF, but it is also influenced by the ease with which translation is initiated, elongation occurs, and termination takes place. Quantification of the association of polysomes with different HCV subgenomic RNA molecules was performed to examine the negative effect of the CRE region on translational efficiency.

**Figure 2:**
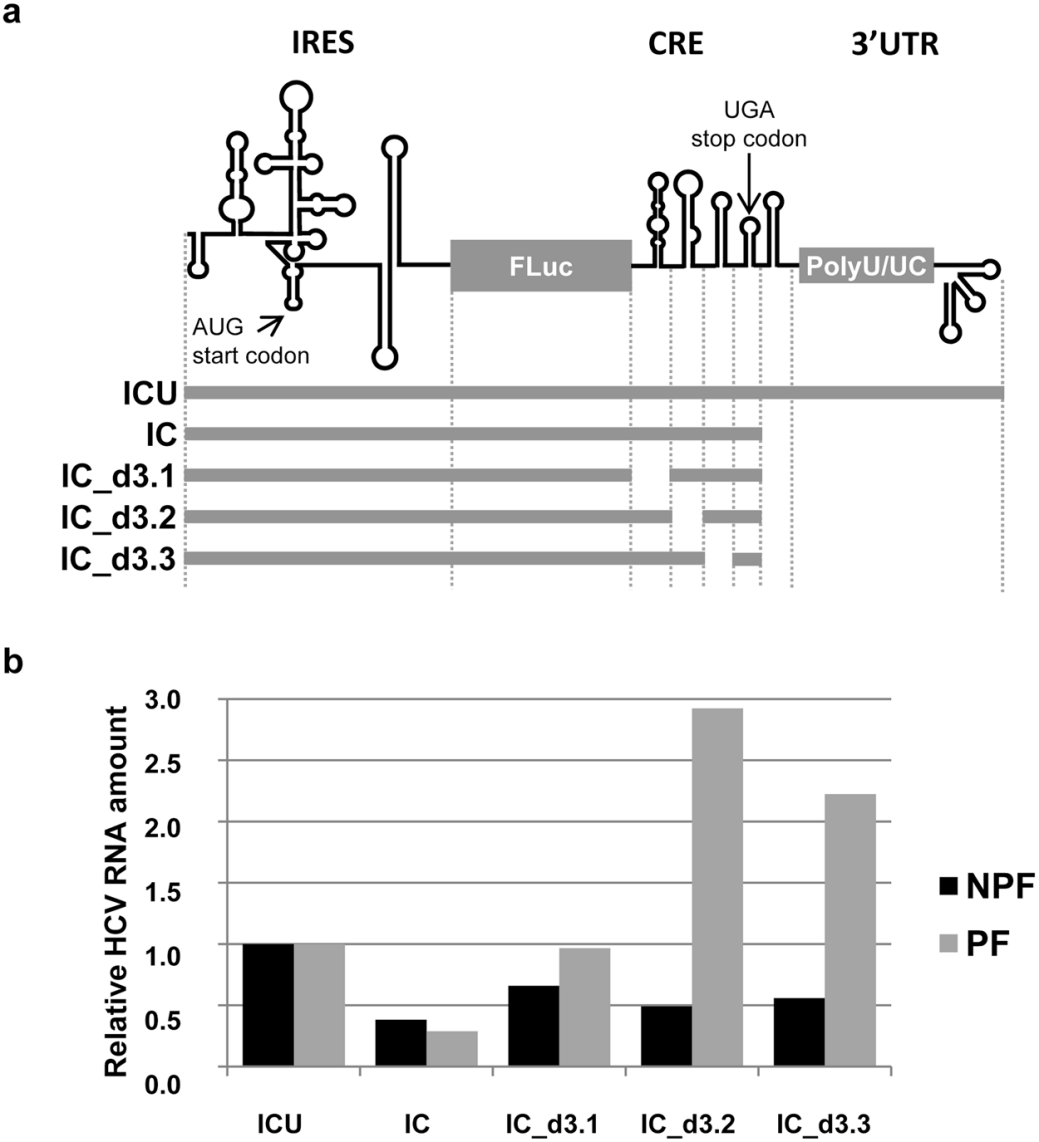
Inhibition of ribosomal assembly by the CRE. (**a**) Diagram of the HCV transcripts harbouring the IRES and the CRE regions used in the polysome profiling assays. (**b**) The CRE region hinders ribosome association with the subgenomic HCV RNA transcripts. Huh-7 cells were transfected with the different RNA constructs shown in (**a**). At 4 h post-transfection, cell lysates were subjected to polysome separation followed by RT-qPCR for quantification of the viral subgenomic transcripts. The histogram represents relative HCV RNA levels normalised to those obtained for the cellular GAPDH mRNA. NPF, non-polysomal fractions; PF, polysomal fractions.

The previously reported transcript ICU^23^ (Fig. 2a) was used to assess translational efficiency via polysome binding assays. The ICU molecule encompasses the HCV IRES region fused to the sequence coding for the FLuc protein, followed by the CRE and the 3′UTR; it therefore meets the minimal requirements for controlling viral translation. In addition, the translationally defective transcript IC, which lacks the 3′UTR (see Fig. 2a and Methods section), allowed the role of the CRE region in polysome formation to be analysed. Huh-7 cells were transfected with HCV subgenomic RNA molecules. Translation was stopped 4 h post-transfection using cycloheximide, and the cells then lysed. Cytoplasmic lysates were fractioned in 15–50% sucrose gradients and specific RNAs detected in each fraction were amplified by RT-qPCR. The non-translatable RNA667^22^ (see Methods) and cellular GAPDH mRNA were chosen to normalise RNA levels and to identify both the non-polysomal and polysomal fractions (NPF and PF respectively; see Methods).

The levels of the viral subgenomic transcript ICU were comparable in terms of their NPF and PF (Fig. 2b). This suggests the ICU molecule to be associated with a suboptimal translational yield. Compared to ICU, a reduction in ribosome binding of ~65% was seen for the IC molecule, both for the NPF and PF (Fig. 2b). This corroborates previous findings showing the inhibitory effect on translation exerted by the CRE^11^. The RNA transcripts derived from the IC molecule with in-phase deletions of the CRE domains - the so-called IC_d3.1, IC_d3.2 and IC_d3.3 transcripts^11^ (Fig. 2a) – showed a range of improvement in polysome association compared to the IC molecule (Fig. 2b). Deletions of the 5BSL3.2 and 5BSL3.3 domains led to a 2–3 fold increase in the PF compared to the ICU control transcript, while deletions of the 5BSL3.1 domain only recovered the translational efficiency of the parental ICU. These data confirm that the CRE domains are key elements in the regulation of translation.

### The CRE region associates with 40S ribosomal subunits *in vitro*

**Figure 3:**
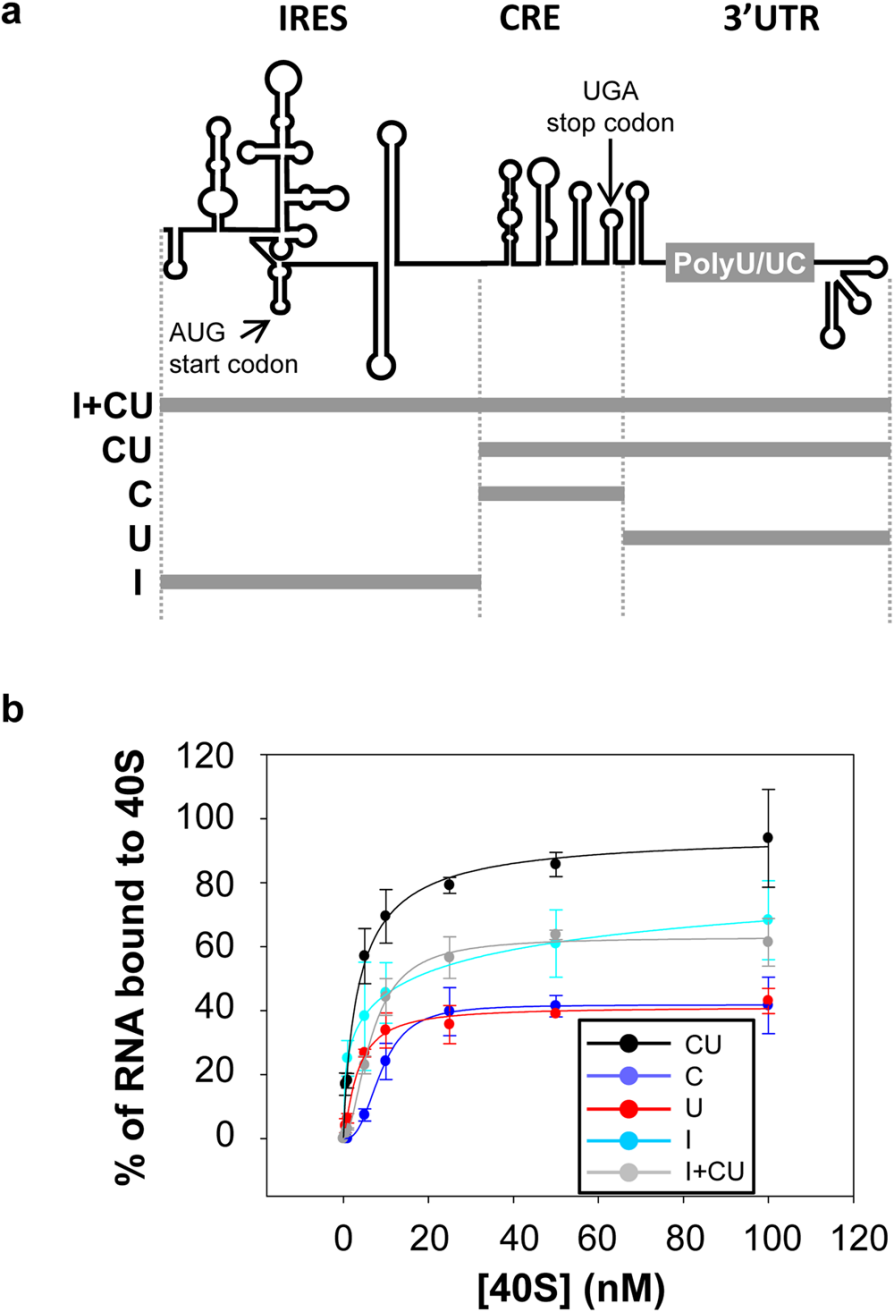
The CRE binds the 40S subunit in the absence of any other protein factor. (**a**) RNA constructs used in binding assays. (**b**) The 40S recruitment by the 3′ end of the viral genome is concentration-dependent. The ^32^P-internally labelled transcripts C, U, CU or I + CU were used as probes and challenged with increasing concentrations (0–100 nM) of the purified 40S ribosomal subunits. Complexes were fractioned by differential filter retention assays. Data correspond to the mean ± standard deviation of four independent experiments.

Polysome binding assays showed that the CRE itself hinders the early association of the ribosomes with the HCV IRES region. Since the CRE has been reported to bind ribosomal proteins^24^, it was questioned whether a CRE:40S interaction might occur. Preliminary results from *in vitro* association assays conducted using purified 40S subunit preparations and the transcript named C, which bears the CRE region^22^ (Fig. 3a), provided unequivocal evidence of a direct interaction. Subsequent binding experiments in the presence of trace amounts of the CRE and increasing concentrations of the 40S ribosomal subunit showed that binding proceeded in a dose-dependent and high-affinity manner, with a K_d_ value in the low nM range (Table 1, Fig. 3b).

**Table 1:** Binding constants.

RNA	K_d_ (nM)	B_max_ (%)	Hill coefficient
C	8.87 ± 0.51	41.85 ± 0.17	0.91 ± 0.13
U	1.09 ± 0.04	41.65 ± 0.14	4.74 ± 0.78
CU	3.64 ± 0.63	95.31 ± 6.69	2.71 ± 0.82
I + CU	6.62 ± 0.36	62.01 ± 1.85	1.92 ± 0.23
I	25.15 ± 1.01	68.30 ± 15.84	0.37 ± 0.02

Values are the means of four independent experiments ± the standard deviation. K_d_, dissociation constant; B_max_, final extent of the complex formation.

To evaluate the contribution of the 3′UTR to CRE-mediated 40S recruitment, *in vitro* binding assays were performed with the transcript CU^23^ (Fig. 3a); the latter bears the CRE region fused to the 3′UTR. The results showed a significant improvement (*p* < 0.05) in both the affinity and the yield of the reaction compared to those obtained for the CRE region alone (Fig. 3b and Table 1), most likely due to the presence of additional anchoring sites for the 40S subunit in the 3′UTR region^10^. In good agreement with previous results, the transcript encompassing the 3′UTR, named U (Fig. 3a), was found able to recruit the 40S subunit with a 8-fold increase in affinity over that shown by the CRE, and with similar efficiency (Fig. 3b and Table 1). Moreover, a cooperative behaviour was observed for the construct U, with a Hill coefficient of 4.74 (see Table 1). The biochemical properties of the CU:40S complex should therefore be the consequence of those shown by the CRE and 3′UTR regions on their own (Fig. 3b and Table 1). Interestingly, the shape of the curve suggested cooperative behaviour, with a calculated Hill coefficient of 2.71 (Table 1), thus preserving one of the biochemical properties shown by the 3′UTR. This undoubtedly indicates the existence of different anchoring sites in the viral 3′ end for the 40S subunit. These results demonstrate that the binding of the 40S subunit may occur independently, and in a cooperative manner, at different sites in the 3′ end of the viral genome.

The HCV IRES is also able to directly recruit the 40S subunit in the absence of any other factor, with both high affinity and efficiency^4^. To determine whether the presence of the IRES might influence the binding of the 40S subunit at the 3′ end, additional binding assays were performed with the transcript encompassing the IRES region plus the CRE and the 3′UTR (I + CU; Fig. 3). Interestingly, the presence of the IRES reduced both the binding affinity and efficiency of I + CU (Fig. 3b and Table 1) compared to CU, achieving similar yield of the complex formation to that shown by the IRES region alone (construct I; Fig. 3 and Table 1). This data points to a potential preferred binding site for the 40S subunit located in the IRES region, in the absence of any other cellular or viral factors.

Together, the results suggest that the 40S binding performed by the HCV genome does not occur as an isolated event; rather, connections between regions of the viral RNA control the efficiency of this process and might even determine the precise region in which binding is favoured, most likely in a viral stage-dependent manner.

### The 40S ribosomal subunit has different anchoring sites in the CRE region

**Figure 4:**
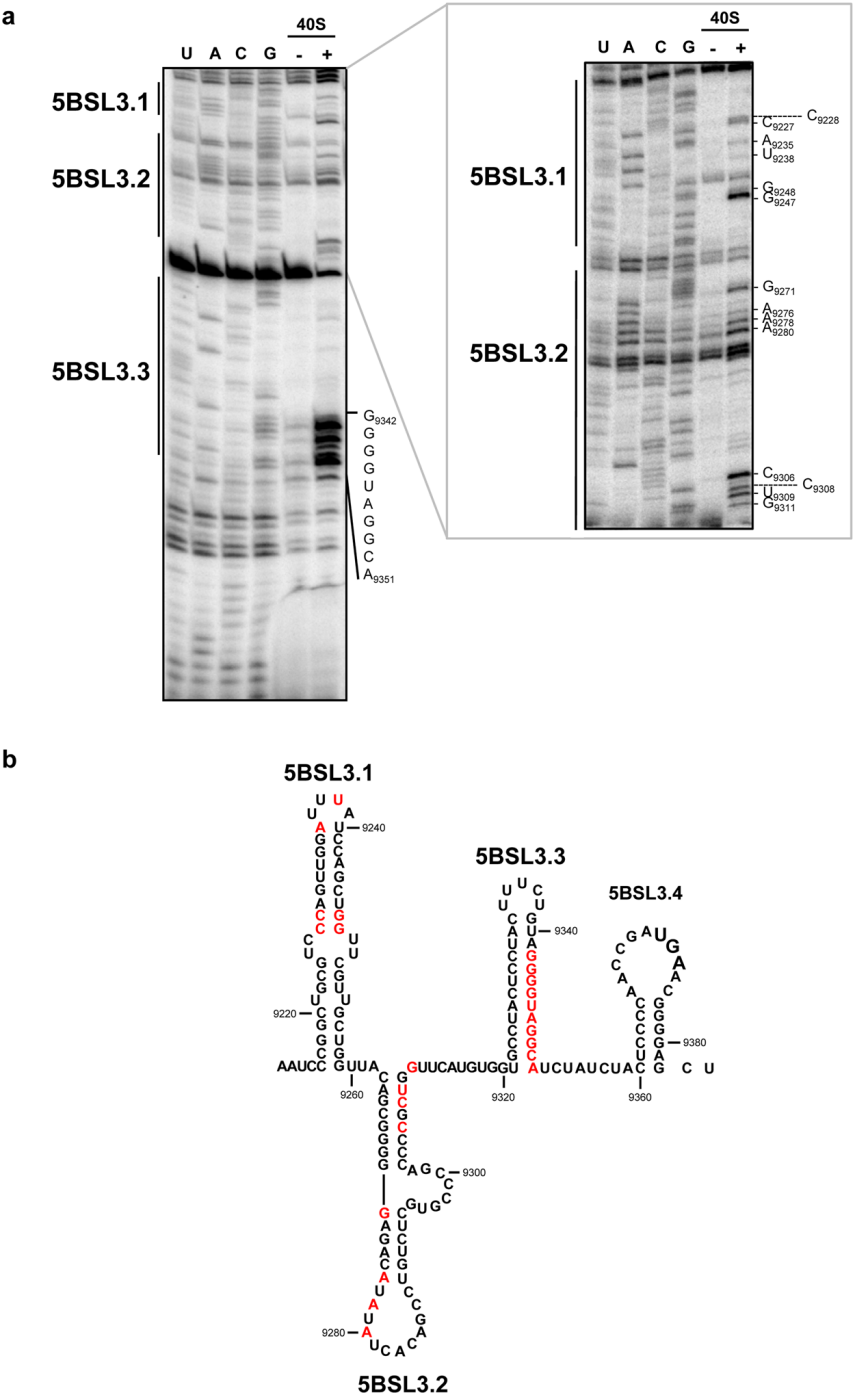
Toeprinting analysis of the CRE:40S complex. (**a**) Representative autoradiographs of the toeprinting assays in the HCV CRE region in the presence of the 40S subunit. Primer extension analysis of the CU transcript were performed with a ^32^P-end labelled oligonucleotide, in the absence (−) or presence (+) of the 40S subunit. cDNA products were analysed in 4%-6% denaturing polyacrylamide gels in parallel with a sequence ladder obtained with the same labelled primer. (**b**) Summary of the results of toeprinting analysis of the CRE region. Nucleotides depicted in red correspond to those identified as 40S-dependent reverse transcription stops. Numbering refers to the nucleotide positions of the HCV Con1 isolate (GenBank accession number AJ238799).

Toeprinting experiments were performed to map the CRE domains involved in the interaction with the 40S subunit. A molar excess of 40S ribosomal subunits was incubated in binding buffer with a transcript bearing the CRE region fused to the first 39 nt of the 3′UTR (CRE + HV)^24^. The RNA was then reverse transcribed using a ^32^P-end-labelled primer complementary to its 3′ end (see Methods). A reaction without the 40S particle was used as a control. The resulting extension products were analysed in high-resolution denaturing polyacrylamide gels with RNA sequencing ladders run alongside to accurately map the cDNA products. The results showed the presence of several specific reverse transcription pauses in the presence of the 40S subunit compared to the extension pattern obtained in its absence (Fig. 4a), suggesting different potential contact sites for the 40S subunit. One of these potential sites occupies a relatively long stretch of nucleotides on the 3′ side of the 5BSL3.3 domain’s stem, from residues G_9342_ to A_9351_ (Fig. 4). This strongly suggests that 5BSL3.3 acts as an anchoring element for the 40S subunit. The increase in the radioactive signal at clear, short-stretch regions within the essential 5BSL3.2 and 5BSL3.1 domains is also noteworthy (Fig. 4a). Taken together, the toeprinting results confirm the association of the 40S particle with the CRE region of the HCV genome, and support the existence of a major recruiting centre in the 5BSL3.3 domain.

**Figure 5:**
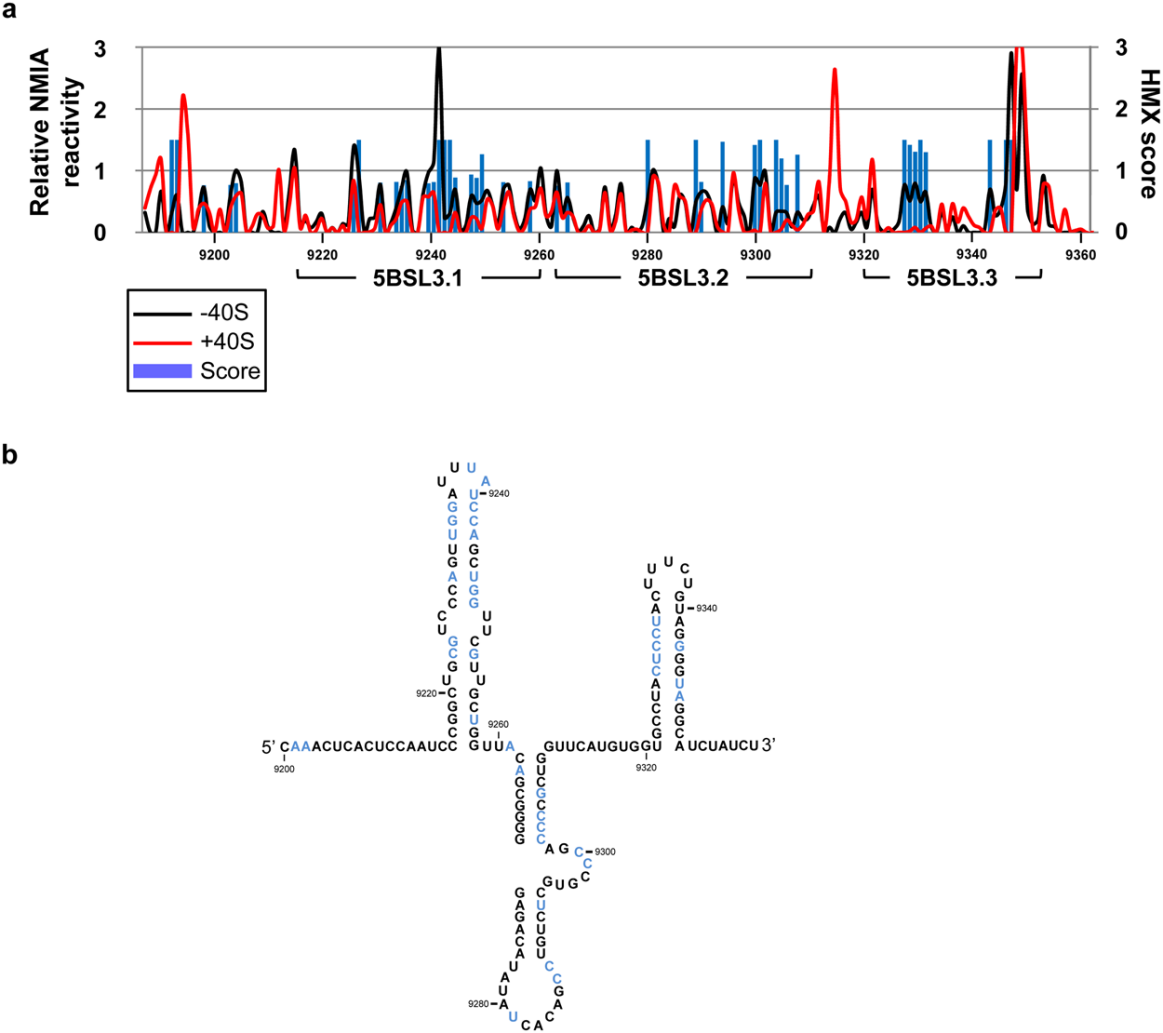
Identification of nucleotides required for CRE:40S complex formation. A molecular interference strategy based on SHAPE chemistry (HMX) was designed to identify those HCV CRE nucleotides involved in the recruitment of the 40S subunit. (**a**) CU construct was modified with NMIA under denaturing conditions and used for binding reactions with a molar excess of the 40S subunit. The free RNA and the CU:40S were fractionated by differential filter retention. Modified positions were detected as stops in a reverse transcription reaction. The line graph shows the NMIA reactivity profile for each of the isolated CU RNA pools, in the absence (−40S, black line) or presence (+40S, red line) of the 40S ribosomal subunit. The overlaid blue histogram shows significant HMX scores (>0.75). HMX scores were calculated from the reactivity profiles of the unbound and complexed CU RNA, as previously described^28^. (**b**) Summary of HMX data, with those nucleotides showing an HMX score for 40S binding of >0.75 depicted in blue. Nucleotide positions are indicated as noted in Fig. 4.

An interference strategy^27^ was next designed to determine the structural requirements at each position for the formation of the CRE:40S complex. For this, the HMX methodology (2′-hydroxyl molecular interference) was followed, using 2′-hydroxyl selective SHAPE chemistry^28^ with the NMIA (N-methylisatoic anhydride) reagent, as previously described^29^ (see Supplementary Fig. 1). NMIA is well-suited for molecular interference applications since it efficiently reacts at high temperature with the specific 2′-hydroxyl group of the RNA backbone, to form a covalent adduct (Supplementary Fig. 1). This reaction accounts, theoretically, in all positions of the RNA under study with similar efficiency. In the analysis of the CRE:40S, it is expected that some of the added adducts will interfere with the proper formation of the complex. Binding reactions were performed subsequent to RNA modification under denaturing conditions, and further to RNA renaturation. The RNA:40S complexes were then partitioned by their filter retention differences, and then eluted. Modified positions were detected as stops by primer extension, as previously described^23^. Those adducts able to prevent the CU:40S complex formation were then identified by comparison of the NMIA reactivity profiles between bound and free CU molecules (Fig. 5 and Supplementary Fig. 1). The resulting reactivity data were cross-related to calculate the HMX score^28^, and therefore identify the residues preferentially (and significantly) modified in the bound RNA (see Methods). In this assay, the CU transcript was used instead of CRE + HV to avoid the loss of information at the 3′ end of the CRE, which would be obfuscated by the outlying fluorescent signal in the shortest extension products.

The results revealed that the structural requirements for the establishment of the CU:40S complex rely on discrete regions of the CRE (Fig. 5). Of note is the significant reduction in NMIA sensitivity (HMX score 1.43 ± 0.09) detected in residues C_9327_-U_9331_, which map to the 5′ flank of the 5BSL3.3 stem. Interestingly, these nucleotides are partially complementary to those mapped by toeprinting at the 3′ side of the 5BSL3.3 stem, confirming 5BSL3.3 as a major binding site for the 40S subunit. From a functional point of view, this finding provides an interesting starting point for investigating the potential role of the 5BSL3.3 domain in HCV translation control.

A number of residues in the apical portion of the stem-loop of domain 5BSL3.1 were also identified as critical partners in the formation of the CRE:40S complex, with an average HMX score of 0.95 ± 0.24 (Fig. 5). This result partially overlaps with the information obtained from the previous toeprinting assays, while providing complementary information regarding the structural requirements of 5BSL3.1 for the formation of the CRE:40S complex. According to the HMX results, the apical portion of 5BSL3.1 must assume a flexible conformation for the recognition of the 40S ribosomal subunit (Fig. 5).

Finally, nucleotides in the 3′ portion of the 5BSL3.2 domain also showed a significant reduction in NMIA reactivity in the presence of the 40S subunit when compared to the unbound CU RNA (mean HMX score 1.03 ± 0.39). Indeed, several of these nucleotides belonged to the essential bulge of 5BSL3.2 (Fig. 5). This result fits well with the toeprinting results, and confirms the importance of domain 5BSL3.2 in 40S binding and, therefore, in HCV translation regulation, as previously proposed^11^.

In summary, these observations demonstrate that the CRE region binds the 40S ribosomal subunit at discrete and precise nucleotides in a conformation-dependent manner. In addition, they imply a new role for the highly conserved 5BSL3.3 and 5BSL3.1 domains as sequestering centres for the cellular translation machinery.

### The CRE:40S interaction depends on specific proteins

**Figure 6:**
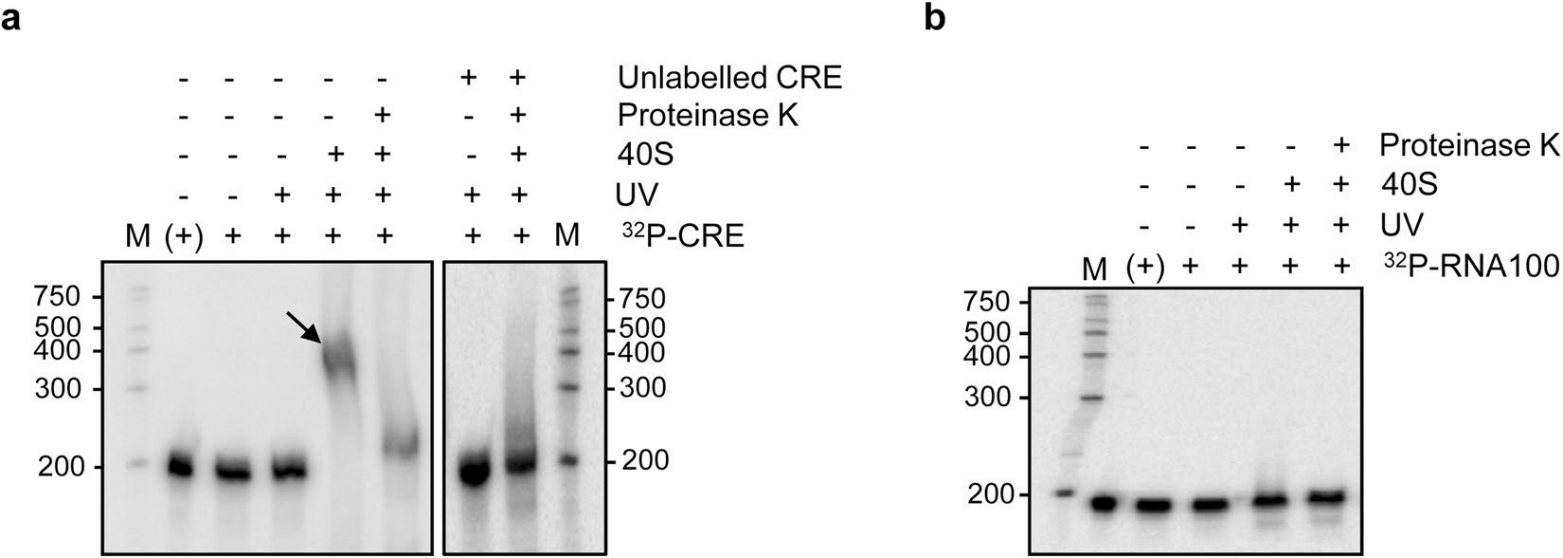
The CRE:40S interaction is protein-dependent. A molar excess of the 40S subunit was incubated with the ^32^P-internally labelled CRE + HV RNA (**a**) or the non-related transcript RNA-100 (**b**). Subsequent cross-linking by UV light radiation led to the formation of a stable complex (indicated by an arrow; compare panels a and b), degradable by proteinase K. Competition with unlabelled RNA CRE + HV (**a**) promoted an increase in the radioactive signal corresponding to the free RNA. M = RNA Century^TM^ marker plus; (+), RNA in the absence of cross-linking buffer.

To identify the 40S components that promote the binding to the CRE region, ^32^P-internally radiolabelled C transcript (Fig. 3a) was subjected to cross-linking by UV irradiation in the absence or presence of the 40S subunit (see Methods). Following exposure to UV light, a slowly migrating product was noted during denaturing polyacrylamide gel electrophoresis in the presence of 40S compared to the transcript C alone (Fig. 6a) - agreeing well with the formation of the CRE:40S complex. Interestingly, protease treatment of the cross-linked CRE:40S complex led to the appearance of a reduced molecular weight product that showed an electrophoretic mobility similar to that of the C molecule (Fig. 6a). This suggests a direct interaction between the CRE and the protein components of the 40S particle rather than with the rRNA. To check this, the experiment was performed in the presence of a molar excess of the non-labelled transcript C; the result was a lack of detectable complex (Fig. 6a). As expected, the non-related RNA-100, which encompasses a fraction of the multiple cloning site of the PBS SK + plasmid (see Methods section), was unable to form any stable complex with the 40S subunit, even after UV irradiation (Fig. 6b and Supplementary Fig. 2). Together, these results demonstrate that CRE binds the 40S ribosomal subunit in a specific manner.

To determine the ribosomal proteins directly involved, label-transfer assays coupled with a proteomic identification strategy were performed. The ^32^P-internally labelled C RNA molecule (Fig. 3a) was incubated in cross-linking buffer with a molar excess of the 40S subunit. The resulting complex was cross-linked by UV irradiation and further treated with RNase A. The reaction products were resolved by SDS-polyacrylamide gel electrophoresis and isolated for subsequent analysis by mass spectrometry. MS/MS ion searching using the Mascot search engine revealed two candidate ribosomal proteins^30^: ribosomal proteins SA (RPSA) and 29 (RPS29).

Taken together, these results indicate that the CRE:40S interaction is protein dependent.

## Discussion

The CRE region is a highly conserved element of the HCV genome critical for viral translation and replication. How these processes are coordinated and regulated, however, has been difficult to answer. The present work provides evidence that the CRE sequesters 40S ribosomal particles, which might be related to the negative regulation exerted by it on IRES-dependent translation, and the concomitant increase in viral RNA replication.

Our group earlier reported that the HCV CRE region operates as an HCV IRES translation repressor, with 5BSL3.2 the main domain involved^11^. It was proposed that the establishment of the long-range interaction IIId-5BSL3.2 might interfere with the proper recruitment of the 40S ribosomal subunit within the HCV IRES region^11^, resulting in the initiation of protein synthesis being blocked. The results of the present polysome binding assays (Fig. 2b) and the ribosomal sedimentation profiles (data not shown) reveal that a defect in the formation of the productive 80S translational complex is the most likely cause of this repression^11^. The appearance of the latter defect is directly dependent on the activity of the three essential domains composing the CRE region, two of which 5BSL3.1 and 5BSL3.3 - have had unclear roles in the progression of the viral cycle. The present work provides the first clues regarding the activity of these domains in the regulation of HCV protein synthesis.

The *in vitro* binding assays and further mapping studies revealed a direct interaction between the CRE RNA and the 40S particle in the absence of any other factor, supporting the assumption that different domains of the CRE cooperate to form complex (Figs 3b–5). It is noteworthy the relatively low binding yield of the constructs C or U with the 40S subunit (Fig. 3b and Table 1), most likely due to the existence of different conformational isoforms, some of them incompatible with the recruitment of the 40S particle. Further, it seems also likely that the resulting complexes are not stable enough under the experimental conditions tested, thus leading to an underestimation of the binding yield for these RNA molecules, C and U, with the 40S subunit. This is supported by the observation that those conditions assessing the complex stabilization, as UV-mediated cross-linking, achieve a 100% binding yield (Fig. 6a).

Interestingly, the binding of the 40S subunit to the CRE does not compete with the previously reported association of the same subunit with the HCV 3′UTR^10^. Rather, both the CRE and the 3′UTR function as independent elements, their contributions both going towards the high complex-formation efficiency achieved (K_d_ value in the low nM range) (Fig. 3b and Table 1). This result implies that, in the context of the CU transcript, the CRE-3′UTR contact would be absent in the presence of the 40S subunit^10,16^. Interestingly, the presence of the IRES reduced the affinity and the recruitment rate of the 40S ribosomal subunit compared to that shown by the whole 3′ end on its own (i.e., the CU transcript; Fig. 3b and Table 1). This suggests that the 40S binding by the HCV genome does not occur as an isolated event. Rather, long-distance connections between different regions of the viral RNA must control the efficiency of this process, and might even determine the precise region – IRES, CRE or 3′UTR - where binding occurs. This would influence the control of progression from one stage of the viral cycle to the next.

The present data also provide important clues about the functionality of the 5BSL3.1 and 5BSL3.3 domains. While these domains have been identified as conserved units (both in terms of sequence and structurally) at the 3′ end of the HCV ORF^14,15^, their role in infection has been unclear. The classic toeprinting assays and the improved molecular interference strategy used in the present work revealed the involvement of the central part of the stem of 5BSL3.3 as a recruiting centre for the 40S subunit (Figs 4 and 5). In addition, the apical portion of the 5BSL3.1 domain would appear to be a key structural element for the association of the 40S subunit; it might also operate as a target domain for the 40S subunit (Figs 4 and 5). Unfortunately, the present results do not discriminate between the 5BSL3.1 and 5BSL3.3 domains as to which provides the major anchoring sites. However, the results of the polysomal profiling experiments, those of the toeprinting assays, and those of the molecular analyses, together suggest 5BSL3.3 to make a contribution towards the association of the 40S subunit.

**Figure 7:**
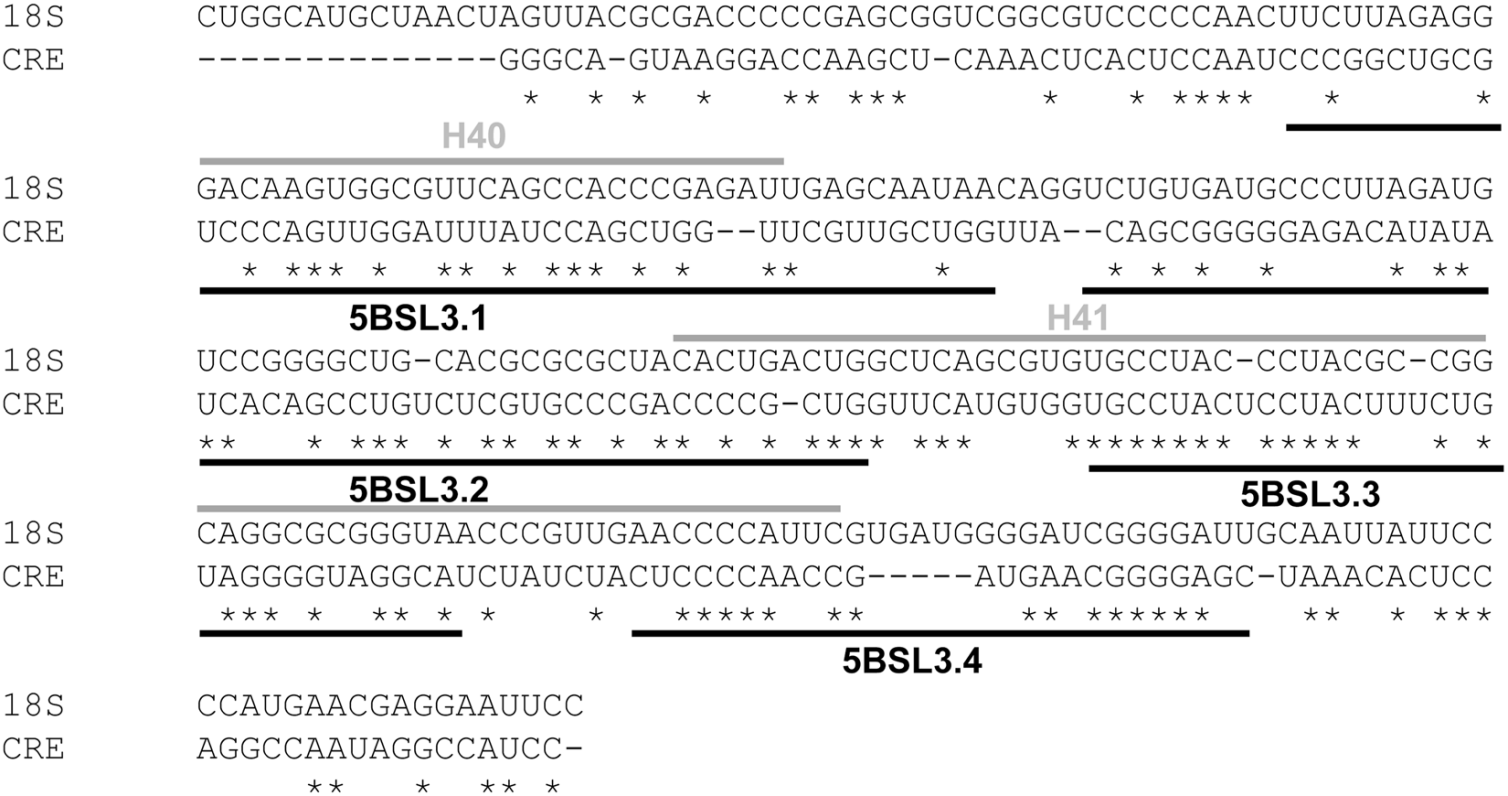
The HCV CRE shows homology with the target sequence for RPSA in the rRNA 18S. The figure shows the multiple alignment of sequences corresponding to the HCV CRE region and the H40-H41 domains of the rRNA 18S, using ClustalW software^46^. The solid black lines denote the CRE, while the H40 and H41 domains from the rRNA 18S are depicted by grey lines.

The anchoring of the 40S subunit to the CRE is dependent on certain ribosomal proteins, among which preliminary analyses include RPSA and RPS29. Like many other ribosomal proteins, RPSA and RPS29 interact with rRNA 18S. Sequence alignment of the CRE and rRNA 18S revealed strong homology between the 5BSL3.1 and 5BSL3.3 domains and the H40 and H41 stem-loops of rRNA 18S (Fig. 7); the latter are the natural anchoring sites for the RPSA protein, but also for ribosomal factor S16^31–33^. This observation, together with the finding that the stem of the 5BSL3.3 domain is a major anchoring site for the 40S subunit, suggests an unknown role for 5BSL3.3 as a manager of translation.

RPS29 locates to the interface between the 40S and 60S ribosomal subunits, close to the initiator AUG in the mRNA, following the formation of the 80S translational complex^31^. It recognises the internal asymmetrical loop of the H23 stem-loop of rRNA 18S^34^, though it seems to show a certain promiscuity with other RNA transcripts that can be targeted by RPS29 and modulated by this protein^34^. According to the present toeprinting and molecular interference results, the bulge of the 5BSL3.2 domain, and the internal loops of the 5BSL3.1 domain, are feasible targets for RPS29. Studies to confirm the involvement of both RPSA and RPS29 in the formation of the CRE:40S complex are currently underway.

It is noteworthy that both RPSA and RPS29 have been identified in HCV IRES-bound complexes by following different proteomic strategies^25,26,35,36^. The sequestration of these factors by the CRE might be used as a strategy to interfere with viral protein synthesis. This idea is reinforced by the observation that the affinity of the HCV IRES for RPSA-deficient 40S subunits is reduced^37^, providing firm support for the idea that the recruitment of RPSA helps regulate HCV IRES-dependent translation.

In addition to their roles as ribosomal components, both RPSA and RPS29 act as soluble or receptor proteins in the cell. In this context, RPSA is a laminin-binding protein with implications in tumour invasion. It can also operate as a potential receptor for the envelope proteins of different flaviviruses, such as West Nile or Dengue virus^38,39^. RPS29, in contrast, enhances the tumour suppressor activity of the Ras-related protein 1A^40^. These functions provide clues about the theoretical consequences that the sequestering of RPSA and RPS29 by the CRE might entail, such as avoiding co-infection with other members of the *Flaviviridae* family or the induction of hepatocarcinoma. This points to new roles of the CRE region beyond the control of viral translation and replication.

Taken together, the present results support a working model in which the translation inhibition effect mediated by the CRE can be envisioned as a multifactorial process involving not only the 5BSL3.2 domain as previously proposed^11^, but also 5BSL3.1 and 5BSL3.3. Three different regulatory pathways could define such an inhibitory mechanism: (i) first, the interaction IIId-5BSL3.2 would impair HCV IRES-dependent translation by impeding the recruitment of the 40S subunit by subdomain IIId^11^; (ii) second, the high affinity binding of the 40S subunit to the CRE region, either at the 5BSL3.1 or the 5BSL3.3 domains, or both, might prevent active 80S ribosomes from penetrating the 3′ end of the viral ORF. This might promote the stalling of polysome production and impede the proper re-loading of the 40S subunit at the 5′ end mediated by the 3′UTR^10^. This defect in ribosome penetration would be enhanced by the presence of highly stable hairpins (formed by 5BSL3.1 and 5BSL3.3) that cannot be bypassed by the 80S translational complex^41^. A major consequence would be an intracellular increase in inefficiently translated viral RNA genomes, but which could be used as replication templates; iii) finally, the sequestering of translation factors by the CRE might be considered a feasible means of interfering with HCV protein synthesis. Such a hypothesis is supported by previous reports describing the binding of the translation machinery proteins to the CRE region^24^. Further work is needed to clarify whether these *in vitro* findings can be exported to an *ex vivo* infection model.

In summary, the present work identifies a high affinity interaction mediated by the CRE and the 40S ribosomal subunit, confirming the multivalent role of the CRE region as a regulator of replication and as a recruiting centre for the translation machinery. The proposed interaction supports the idea that CRE-mediated translation is a multifactorial process involving different capacities and functionalities of the CRE, and suggests new and unsuspected roles for 5BSL3.1 and 5BSL3.3 as switching partners that manage the transitions between the different steps of the HCV cycle.

## Methods

### DNA templates and RNA synthesis

The DNA template constructs for IC, ICU, IC_d3.1, IC_d3.2, IC_d3.3, C, CRE + HV, U, I + CU and CU RNA were obtained by amplification using specific primers as previously described^11,22,24^.

The template for RNA-100 was generated by endonuclease digestion of the pBS SK(+) plasmid with *Xba*I. RNA667 was obtained as previously reported^22^.

RNA molecules were *in vitro* synthesised using the TranscriptAid T7 High Yield Kit (Thermo Fisher Scientific), following the manufacturer’s instructions. The quantity of RNA produced was determined by UV spectrophotometry (A260); protein and carbohydrate/phenolic contamination was assessed via the A260/A280 and A260/A230 ratios respectively. The integrity of the transcripts was tested by denaturing agarose gel electrophoresis. Internally radiolabelled RNA molecules were synthesised as previously described^22^.

### Cell culture and transfection

Human hepatoma Huh-7 cell monolayers were maintained in Dulbecco’s modified Eagle medium (DMEM) supplemented with 10% heat-inactivated foetal bovine serum (Invitrogen) and 1 mM sodium pyruvate (Sigma), at 37 °C in a 5% CO_2_ atmosphere.

Cell transfections were performed essentially as previously reported^11^. Briefly, 48 h before transfection, 500,000 Huh-7 cells were seeded onto 10 cm-diameter plates to reach 90% confluence. 5 µg of the constructs IC, ICU, IC_d3.1, IC-d3.2 or IC_d3.3 and 1 µg of the RNA667 were then mixed with 50 µl of Opti-MEM® (Invitrogen) and 2 µl of transfection reagent (TransFectin^TM^; Bio-Rad) and added to cell cultures. Translational efficiency was analysed by polysome profiling.

### Purification of 40S ribosomal subunits

40S ribosomal subunits were isolated from freshly prepared Huh-7 S10 cell lysates as previously reported, with slight modifications^42^. Briefly, S10 fractions from ~4 g of Huh-7 cells were prepared as described^24^. Ribosomes were then loaded onto 3 ml of a 1 M sucrose cushion in buffer A (Tris-HCl, pH 7.6, 20 mM; DTT, 2 mM; MgCl_2_, 6 mM; KCl, 0.5 M) and precipitated by ultracentrifugation at 40,000 rpm and 4 °C in a Beckman 70.1 Ti rotor for 4 h. The resulting pellet was resuspended in buffer B (Tris-HCl, pH 7.6, 20 mM; DTT, 2 mM; MgCl_2_, 6 mM; KCl, 150 mM) to a concentration of 50–150 A_260_ units. This suspension was incubated with 4 mM puromycin for 10 min at 4 °C, following by incubation at 37 °C for 30 min. Salt concentrations were then increased by the addition of 0.5 M KCl to release the translation initiation factors bound to the ribosomal subunits. The ribosomal subunits were then loaded onto a continuous 10–45% linear sucrose gradient in buffer A and resolved by ultracentrifugation in a Beckman SW40 rotor device at 28,000 rpm overnight. Fractions of 500 µl were collected from the top of the gradient and concentrated using Amicon® Ultra-2 30 kDa. The concentration of the 40S subunits was calculated by UV spectrometry measurements as 1 A_260_ = 30 pmol/ml.

### Polysome profiling

Transfected Huh-7 cells were treated with cycloheximide (0.1 mg/ml from Sigma-Aldrich) at 37 °C for 20 min and then washed twice with PBS supplemented with cycloheximide (0.1 mg/ml). The cells were scraped off and resuspended in ice-cold PBS in the presence cycloheximide (0.1 mg/ml). This suspension was centrifuged at 800 × *g* for 5 min at 4 °C, and the pellet lysed in 2 volumes of polysome lysis buffer (PLB; Tris-HCl, pH 8.0, 10 mM; NaCl 140 mM; MgCl_2_, 1.5 mM; NP-40, 0.5%; DTT, 20 mM; PMSF,1 mM; cycloheximide, 0.1 mg/ml, protease inhibitor cocktail [Roche] and Invitrogen™ Ambion™ ANTI-RNase, 40 U/ml). Cell lysate was centrifuged at 11,000 × *g* for 10 min at 4 °C, to pellet nuclei and debris. The supernatant was overlaid onto a linear 10–50% sucrose gradient in buffer A (Tris-HCl, pH 7.5, 20 mM; NaCl, 100 mM; MgCl_2_, 5 mM). Gradients were centrifuged for 2 h at 190,000 × *g* in a Beckman SW40 swinging bucket rotor apparatus at 4 °C. 1 ml fractions were collected from the top of each gradient and supplemented with a fixed amount (100 ng) of the non-related and non-translatable RNA667 transcript, used as internal control for the subsequent RT-qPCR steps. Fractions were then subjected to RNA extraction with TRIzol reagent (Thermo Fisher Scientific), following the manufacturer’s instructions. RNA integrity was monitored by gel electrophoresis in a 1% agarose gel and quantified by densitometry. The fractions showing a 28S/18S ratio of ≥1.6 corresponded to those bearing polysome-bound RNA. The distribution of the specific RNA molecules containing the HCV subgenomic constructs was determined by RT-qPCR and normalised to that obtained for the GAPDH mRNA, essentially as previously described^43^ with minor modifications. RT-qPCR was additionally performed for the RNA667 and referred to that obtained for the GAPDH mRNA, with the aim of corroborating that RNA extraction and precipitation processes accounted with similar efficiency for all the fractions tested. Briefly, 500 ng of RNA were hybridised with 3 µg of random primers by denaturation at 95 °C and cooling on ice. MultiScribe reverse-transcriptase was then added to a final concentration of 0.5 U/μl and cDNA synthesis performed in the presence of dNTPs (0.5 mM) and the buffer supplied by the manufacturer. The extension proceeded in two steps: slow extension at 16 °C for 30 min, followed by rapid extension at 37 °C for 30 min. The reverse transcriptase was heat-inactivated at 85 °C for 5 min. One sixth of the cDNA mix volume was employed for qPCR with the SsoFast™ Evagreen^®^ supermix (Bio-Rad) and amplified over 40 cycles with specific oligonucleotides targeting the IRES region (C-149 and C-342)^44^ or the RNA667^24^.

### Binding assays

Binding assays were performed with the aim of calculating the dissociation constant values (K_d_) for the HCV RNA:40S subunit. For this, the ^32^P-internally radiolabelled RNA molecules C, CU, U and I + CU were heated at 95 °C for 2 min and then cooled to 4 °C. A concentration of 0.1 nM of the RNA constructs were then incubated with increasing concentrations of the purified 40S subunits (0–100 nM) in binding buffer (Tris–HCl, pH 7.4, 20 mM; K(AcO), 100 mM; KCl, 200 mM; Mg(AcO)_2_, 2.5 mM; DTT, 1 mM) for 30 min at 37 °C. The reactions were then loaded onto a filter sandwich composed of 0.45 µm nitrocellulose filters (GE Healthcare Life Science) able to retain the ribonucleoprotein complexes; these were placed above nylon filters (GE Healthcare Life Science) to trap any RNA not captured by the nitrocellulose membrane. Both nylon and nitrocellulose membranes were pre-soaked in binding buffer and assembled in a dot-blot apparatus. Samples were diluted up to 100 µl with binding buffer and were directly added to the filters under vacuum. Filters were then dried and scanned in a Phosphorimager (Storm820, GE Healthcare) and quantified with Image Quant 5.2© software (GE Healthcare). K_d_ values were calculated using Sigma Plot 8.02© software according to the equation y = y_0_ + (B_max_·x^n^)/(K_d_^n^ + x^n^), where y is the percentage of complexed RNA, B_max_ is the binding yield, x is the concentration of the target RNA, K_d_ is the dissociation constant and n is the Hill coefficient.

### Toeprinting

For each 10 µl reaction volume, a mixture containing the CU RNA (1 pmol) and the ^32^P-end labelled oligonucleotide P4^13,22^ (2 pmol) was denatured and refolded by heating at 95 °C for 2 min followed by slow cooling to 37 °C for 15 min to minimise non-specific interactions. Purified 40S subunits (200 nM) were then added to the mixture and binding reactions allowed to proceed in the presence of binding buffer for 30 min at 37 °C. The control reaction involved substituting the 40S subunit for an equivalent amount of a non-related RNA (RNA667). For subsequent reverse transcription, of SuperScript III reverse transcriptase (200 U from Thermo Fisher Scientific) and dNTPs (0.5 mM) were incubated in a 20 µl reaction volume with the reverse transcriptase buffer provided by the supplier. Extension was allowed to proceed for 30 min at 37 °C and stopped by snap cooling on ice. The reaction products were resolved by high-resolution denaturing polyacrylamide gel electrophoresis (4–6%), dried, scanned in a Phosphorimager (Storm 820, GE Healthcare), and quantified using Image Quant 5.2© software (GE Healthcare).

### 2′-hydroxyl molecular interference (HMX) assays

HMX analysis was performed essentially as previously described^28,29^, with minor modifications. Briefly, the RNA construct CU (5 pmol) was subjected to chemical modification under denaturing conditions (heating at 95 °C) in the presence of 100 mM Hepes pH 8.0 and 25 mM of the modifying reagent NMIA [N-methylisatoic anhydride] for 3 min). The reactions were then cooled on ice and the modification protocol repeated twice. The final reaction volume (20 µl) was achieved at every modification step by replacing the evaporated water. The modified RNA was ethanol-precipitated and the final quantity monitored by UV spectrophotometry (A_260_). Complex formation between the CU molecule and the 40S subunit was accomplished at a molar ratio of 1:5 in binding buffer for 30 min at 37 °C. This complexed RNA-CU was separated from the unbound CU molecules by differential filter retention assays, as described above. RNA molecules were passively eluted from the filters for 3 h at 4 °C and subjected to phenol:chlorofom extraction and ethanol precipitation. NMIA modifications were detected by primer extension and the cDNA products analysed by capillary electrophoresis, as previously described^20^. HMX scores were calculated as previously reported^28,29^.

### UV cross-linking and pull-down experiments to identify ribosomal proteins directly interacting with the CRE region

RNA denaturation and complex formation were performed under similar conditions to those described for the binding assays. Briefly, 10 nM of the internally ^32^P labelled RNA C was incubated with a 5-fold molar excess of the purified 40S subunit in cross-linking buffer (Hepes/NaOH, pH 7.6, 100 mM; DTT, 10 mM; MgCl_2_, 3 mM; KCl, 400 mM; tRNA 50 ng/µl; glycerol, 5%; Invitrogen™ Ambion™ ANTI-RNase, 10U), at 37 °C for 30 min. In parallel, C:40S complex formation was completed with a 5-fold molar excess of the non-labelled RNA C. Control association reactions were also performed with RNA-100, a non-related RNA that does not bind the 40S subunit. Reactions were then exposed to 365 nm UV light for 15 min using a CL-1000 Ultraviolet Crosslinker (UVP). Samples were placed in a cool metal block to prevent them from overheating. The RNA-protein interaction was verified by adding 1 µg of proteinase K in 0.5% SDS and further incubation for 30 min at 37 °C. Samples were resolved in 6% denaturing polyacrylamide gels, which were dried and scanned as previously described^45^.

To identify those ribosomal proteins directly targeting the CRE RNA, the cross-linked binding reactions were treated with RNase A 50 ng/µl and loaded on 14% denaturing SDS polyacrylamide electrophoresis gels. Products corresponding to every RNA-protein complex were cut out and digested with trypsin overnight. Peptides were purified in 0.2% TFA and 30% acetonitrile, crystallised in an “AnchorChip” plate using 0.7 mg/ml CHCA as a matrix, and analysed in an UltraXtreme mass spectrometry MALDI-TOF/TOF device (Bruker). Proteins were identified using the MASCOT search engine, checking against the Swiss-Prot database and allowing for 1–2 trypsin missed cleavages. Only proteins with a Mascot Score of over −10xLog(P) were contemplated (where P is the probability that the observed match is a random event).

## Electronic supplementary material

Supplementary Information
