# Supplementary material for: The HCV genome domains 5BSL3.1 and 5BSL3.3 act as managers of translation
Source: Sci Rep. 2018 Oct 31;8:16101. doi: 10.1038/s41598-018-34422-7 (PMC6208389; doi:10.1038/s41598-018-34422-7)

SUPPLEMENTARY INFORMATION

The HCV genome domains 5BSL3.1 and 5BSL3.3 act as managers of translation

Cristina Romero-López[‡*], Pablo Ríos-Marco[‡], Beatriz Berzal-Herranz and Alfredo Berzal-Herranz[*]

Instituto de Parasitología y Biomedicina López-Neyra, (IPBLN-CSIC), Av. del Conocimiento 17, 18016, Armilla, Granada, Spain

[‡] These authors made equal contributions to this paper

[*] Correspondence: Cristina Romero-López and Alfredo Berzal-Herranz

Email: cristina_romero@ipb.csic.es; aberzalh@ipb.csic.es

Supplementary Figure 1

General scheme of the molecular interference assay using SHAPE chemistry, performed in the present work.

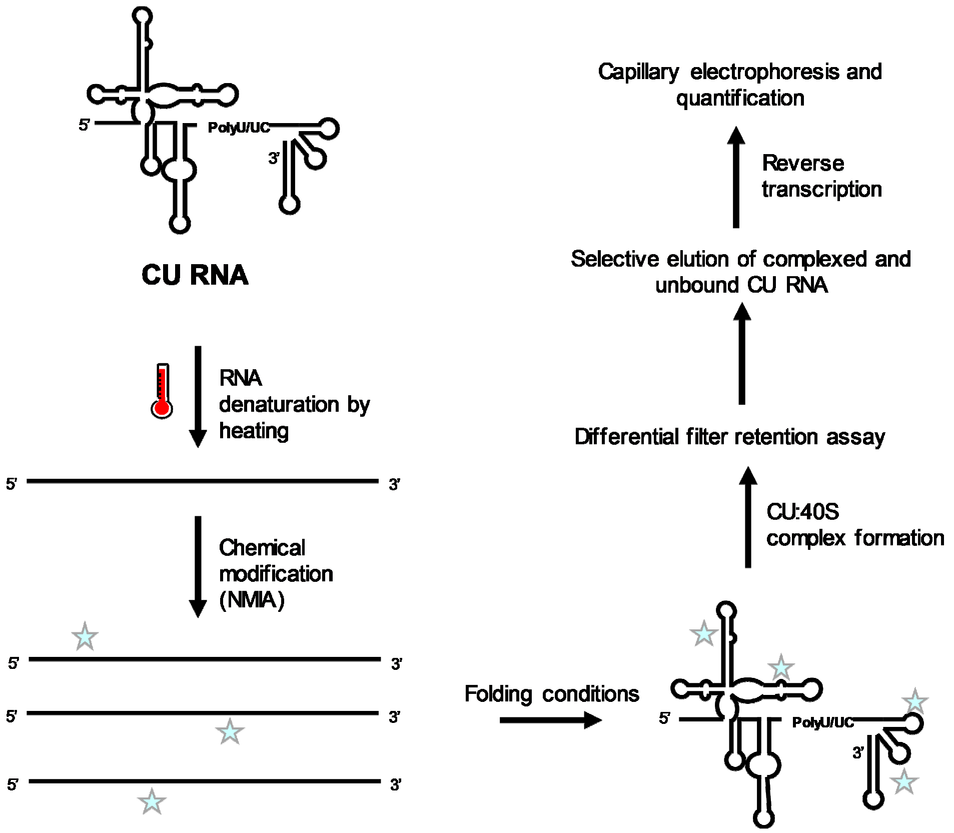

Supplementary Figure 2

Representative double filter retention assay to monitor the 40S recruitment by the non-related RNA-100. Figure shows an autoradiography of nitrocellulose (upper) and nylon (lower) membranes from a titration experiment using a fixed concentration of 0.1 nM of the internally radiolabelled RNA-100 and increasing amounts of the 40S subunit. Quantitative analysis was performed as described in Methods section. The results are shown as % of complex in the bottom row.

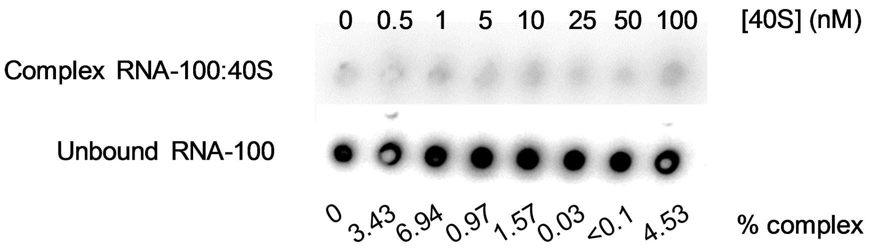

Supplement: Supplementary file 1 — Supplementary Information [file 41598_2018_34422_MOESM1_ESM.pdf]
